# *Perlecan*, a candidate gene for the CAPB locus, regulates prostate cancer cell growth via the Sonic Hedgehog pathway

**DOI:** 10.1186/1476-4598-5-9

**Published:** 2006-03-01

**Authors:** Milton W Datta, Ana Maria Hernandez, Michael J Schlicht, Andrea J Kahler, Amy M DeGueme, Rajiv Dhir, Rajal B Shah, Cindy Farach-Carson, Andrea Barrett, Sumana Datta

**Affiliations:** 1 Departments of Pathology and Urology, Emory University, Atlanta, GA, 30322, USA; 2 Department of Biochemistry and Biophysics, Texas A&M University, 2128 TAMU, College Station, Texas 77843-2128, USA; 3 Department of Pathology, University of Pittsburgh Medical Center, 200 Lothrop Street, Pittsburgh PA, 15232, USA; 4 Department of Pathology, University of Michigan, 1500 Catherine Ave, Ann Arbor, MI, 48109, USA; 5 Department of Biological Sciences, University of Delaware, 304 Wolfe Hall, Newark, DE, 19716, USA

## Abstract

**Background:**

Genetic studies associated the CAPB locus with familial risk of brain and prostate cancers. We have identified *HSPG2 *(*Perlecan*) as a candidate gene for CAPB. Previously we have linked Perlecan to Hedgehog signaling in *Drosophila*. More recently, we have demonstrated the importance of Hedgehog signaling in humans for advanced prostate cancer.

**Results:**

Here we demonstrate *Perlecan *expression in prostate cancer, and its function in prostate cancer cell growth through interaction and modulation of Sonic Hedgehog (SHH) signaling. *Perlecan *expression in prostate cancer tissues correlates with a high Gleason score and rapid cell proliferation. *Perlecan *is highly expressed in prostate cancer cell lines, including androgen insensitive cell lines and cell lines selected for metastatic properties. Inhibition of *Perlecan *expression in these cell lines decreases cell growth. Simultaneous blockade of Perlecan expression and androgen signaling in the androgen-sensitive cell line LNCaP was additive, indicating the independence of these two pathways. *Perlecan *expression correlates with SHH in tumor tissue microarrays and increased tumor cell proliferation based on Ki-67 immunohistochemistry. Inhibition of *Perlecan *expression by siRNA in prostate cancer cell lines decreases SHH signaling while expression of the downstream SHH effector *GLI1 *rescues the proliferation defect. Perlecan forms complexes with increasing amounts of SHH that correlate with increasing metastatic potential of the prostate cancer cell line. SHH signaling also increases in the more metastatic cell lines. Metastatic prostate cancer cell lines grown under serum-starved conditions (low androgen and growth factors) resulted in maintenance of *Perlecan *expression. Under low androgen, low growth factor conditions, *Perlecan *expression level correlates with the ability of the cells to maintain SHH signaling.

**Conclusion:**

We have demonstrated that Perlecan, a candidate gene for the CAPB locus, is a new component of the SHH pathway in prostate tumors and works independently of androgen signaling. In metastatic tumor cells increased SHH signaling correlates with the maintenance of Perlecan expression and more Perlecan-SHH complexes. Perlecan is a proteoglycan that regulates extracellular and stromal accessibility to growth factors such as SHH, thus allowing for the maintenance of SHH signaling under growth factor limiting conditions. This proteoglycan represents an important central regulator of SHH activity and presents an ideal drug target for blocking SHH effects.

## Background

Genetic mapping studies for familial prostate cancer have identified numerous chromosomal regions linked to prostate cancer susceptibility. On chromosome one a genetic association has been demonstrated between clinically significant prostate cancer and the brain tumor glioblastoma multiforme at 1p36 (CArcinoma Prostate Brain, CAPB), suggesting the presence of a common oncogene for these tumors [1-3]. Using bioinformatics based analysis of text mining and gene expression data we have identified candidate genes within the CAPB locus. One of these genes is HSPG2 (Perlecan). Perlecan is a heparan sulfate proteoglycan that is secreted into the extracellular matrix and can bind growth factors [4]. Thus Perlecan can act as a reservoir or modulator of growth factor function. One growth factor associated with Perlecan is Hedgehog [5]. Sonic Hedgehog signaling has recently been shown to be critical for cancer growth and metastasis in multiple tumor types [6]. In a large proportion of prostate cancers high levels of *Sonic Hedgehog *expression is observed along with expression of multiple members of the Hedgehog signaling pathway such as its receptor *Patched1*, downstream transcription factor *Gli1*, and intracellular modulator *Hedgehog Interacting Protein *[7,8]. Activation of the Hedgehog pathway has been detected in metastatic prostate tumors [8,9], and higher levels of pathway activity are associated with the metastatic phenotype [9]. Blocking the Sonic Hedgehog pathway with cyclopamine inhibits proliferation of prostate cancer cell lines [7-9] and primary prostate tumor cell cultures [7]. Treatment of mice with cyclopamine results in the inhibition of tumor xenograft growth in multiple tumor types, including prostate tumors [7,10]. Our bioinformatics analyses [6,7] suggested that genes encoding two components of the Sonic Hedgehog pathway, *Suppressor of Fused *(*Su(fu)*) and *Smoothened*, the target of cyclopamine, lie in chromosomal regions implicated in familial prostate cancer [11,12]. Su(fu) is a negative regulator of pathway activity, thus loss of Su(fu) function would increase Sonic Hedgehog activity. Molecular analyses of prostate tumors revealed that Su(fu) protein is absent in most highly aggressive tumors and somatic truncation mutations in the *Su(fu) *gene have been identified [8] consistent with the hypothesis that *Su(fu) *would act as a prostate tumor suppressor gene by inhibiting Sonic Hedgehog signaling. These studies demonstrate the critical nature of Sonic Hedgehog signaling in tumorigenesis and metastasis. Thus identification of additional mechanisms for the regulation of Sonic Hedgehog signaling in cancer takes on added importance. Here we demonstrate that expression of the candidate CAPB gene *HSPG2 *(*Perlecan*) is present in prostate cancers, up-regulated in aggressive prostate cancers and under poor cell growth conditions, and regulates prostate cancer cell proliferation. In addition, we demonstrate that Perlecan's effects on cell growth are independent of androgen signaling and occur through the binding of Sonic Hedgehog, resulting in modulation of the Sonic Hedgehog-Patched-Gli signaling pathway. This data, along with data linking Perlecan to metastatic tumor environments such a bone matrix [13], presents a general model in which *Perlecan *expression by tumor cells under poor growth conditions enhances their ability to utilize growth factors until their spread to suitable metastatic tumor microenvironments for accelerated growth.

## Results

### *Perlecan *is expressed in and associated with aggressive prostate cancers

**Figure 1:**
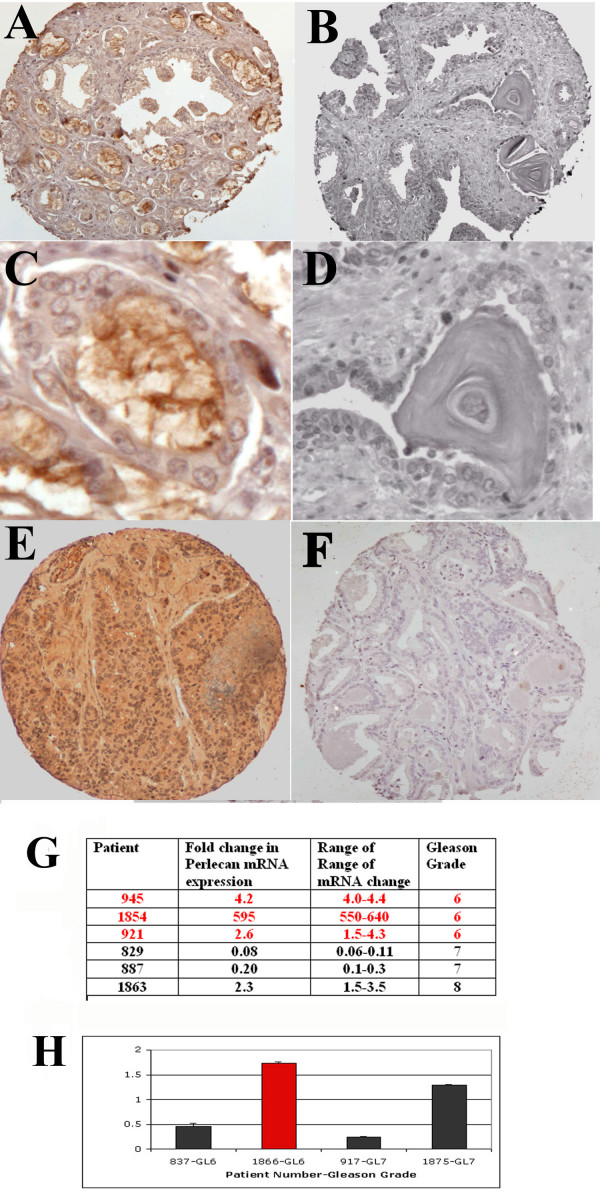
**Perlecan protein levels in human prostate tumors**. Immunohistochemistry of Perlecan protein in prostate cancer (A) and normal prostate (B). Perlecan is present as a secreted protein in the tumor gland lumens (C) but not in the lumens of benign glands or benign corpora amylacea secretions (D). Staining is also seen in metastatic prostate cancer specimens (E). Secondary antibody alone control fails to demonstrate staining (F). All images originally photographed at 400 × magnification. Quantitation of Perlecan mRNA expression by Real Time PCR (G) or protein by digitized dot blot (H) in normal prostate and tumor samples from individual patients presented as fold change in tumor versus normal. Gleason scores for the tumors are listed. Red numbers or columns indicate patients previously shown to have increased expression of *SHH*, *PTCH1 *and *GLI1 *[7].

After identification of *Perlecan *as a candidate gene for the CAPB locus we sought to confirm the presence of Perlecan in primary prostate cancers. Immunohistochemical analysis for Perlecan in prostate cancer tissue microarrays with 600 patient samples demonstrated that Perlecan, a secreted proteoglycan, is present in the lumens of 54% of malignant prostate cancer glands, but not in normal glands (Figure. 1A–D, Table 1). There was a significant increase in Perlecan levels in invasive tumors compared to either benign prostate tissue or the precancerous lesion high grade prostatic intraepithelial neoplasia (HGPIN). In particular *Perlecan *expression was associated with more aggressive tumors, as evidenced by their higher Gleason score (Gleason score 7,8,9 versus Gleason score 5 and 6 tumors). *Perlecan *expression was also significantly associated with increased prostate cancer cell proliferation, as demonstrated by Ki-67 (PCNA) immunohistochemical staining (Table 1). To extend the evaluation of Perlecan we examined *Perlecan *RNA (Figure 1G) and/or protein (Figure 1H) levels in matched benign and tumor samples from 10 individual patients. At the RNA level *Perlecan *was significantly increased in four out of six matched patient tumor and benign prostate samples. Perlecan protein was upregulated in two of four additional patient samples where protein was examined. An examination of the Gleason score for the primary tumor samples revealed that the only Gleason score 8 tumor upregulated Perlecan. These findings correlate with the results from the tissue microarrays (Table 1). Perlecan Ki-67 staining was also evaluated in five of the patient samples, two with low Perlecan, and three with increased Perlecan expression. Immunoblotting demonstrated a direct correlation between increased Perlecan expression and increased Ki-67 levels. These findings matched the immunohistochemical staining results from the tissue microarrays (Table 1). We also examined Perlecan protein expression on tissue microarray samples from patients with primary and metastatic prostate cancer identified at autopsy. In these samples Perlecan expression was upregulated in the primary prostate tumor and metastatic prostate cancer that had spread to the lungs and liver (Figure 1E, Table 1). Perlecan expression was lower in tumor present in lymph nodes or soft tissue metastasis, indicating site specific differences in Perlecan expression in metastatic prostate cancer.

**Table 1:** Immunohistochemical Staining for Perlecan and Co-Localization with Ki-67.

		**Perlecan Negative**	**Perlecan Positive**	
Histology	Tumor	170	203	
	Normal	211	31	p < 0.00005
	Tumor	170	203	
	HGPIN	46	7	p < 0.00005
Clinical Stage	cT2	11	12	
	cT3/4	2	3	N.S.
Tumor Grade	Gleason 6	26	5	
	Gleason 7,8,9	38	23	p = 0.0335
Pathologic Stage	pT1-pT2	35	17	
	pT3	29	11	N.S.
Nodal Status	pN0	18	23	
	pN1	0	1	N.S.
Outcomes	PSA Recurrence	4	5	
	No PSA Recurrence	13	22	N.S.
Vital Status	Alive	26	36	
	Dead	2	5	N.S.

**Perlecan Expression in Metastasis**				

**Metastatic Site**		**Perlecan Negative**	**Perlecan Positive**	**P value vs. Prostate**

Primary Tumor (Prostate)		3	24	
Lymph Node		8	9	p = 0.0073
Soft Tissue		15	18	p = 0.0039
Liver		5	23	p = 0.4781
Lung		3	24	p = 1.000

**Association of Ki-67 (PCNA) Staining with Perlecan Staining**				

**Sample Staining**		**Number of Samples**	**Mean % of Ki-67 positivity**	**Two-tailed ANOVA**

Perlecan positive		143	6.715278	p = 0.0478
Perlecan negative		214	5.028571	

* Tumor grade is presented as Gleason score. Pathologic staging uses the American Joint Commission on Cancer (AJCC) 2002 tumor staging criteria. HGPIN = high grade prostatic intraepithelial neoplasia.

### Basal *Perlecan *expression is highest in an androgen sensitive tumor cell line

**Figure 2:**
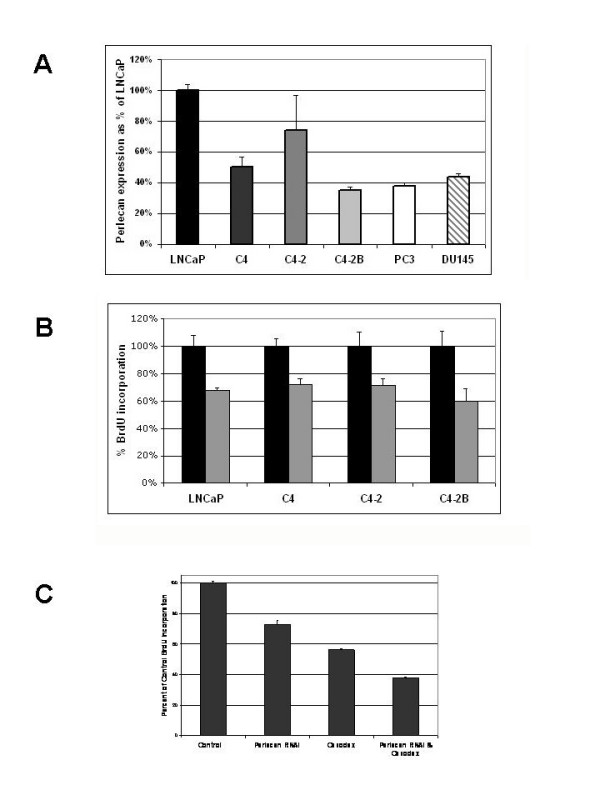
**Perlecan expression and functional analysis in cell lines**. A. Relative Perlecan mRNA levels from Realtime PCR (LNCaP series) and spotted cDNA microarray data (LNCaP, DU145, PC3). All samples presented normalized to LNCaP at 100%. Androgen sensitive: LNCaP. Androgen insensitive: C4, C4-2, C4-2B, PC3, DU145. B. Inhibition by Perlecan siRNA decreases prostate cancer cell proliferation. BrdU incorporation in the LNCaP, C4, C4-2 and C4-2B cell lines. All samples were normalized to control (scrambled siRNA treated) cells at 100%. Black bars represent control samples transfected with scrambled siRNA. Grey bars represent samples transfected with Perlecan siRNA. Error bars represent n = 3 independent samples. C. Additive effect of Perlecan siRNA and androgen blockade on cell proliferation. BrdU incorporation in LNCaP cells after Perlecan siRNA and/or bicalutimide (Casodex) treatment. Control and Casodex alone samples were treated with a scrambled siRNA. p < 0.0001 for comparisons between groups. Error bars represent n = 6 for independent transfections.

Baseline expression of *Perlecan *was examined in the metastatic prostate cancer cell lines LNCaP, DU-145, and PC3. Using analysis of spotted cDNA microarray expression data [14] quantitative Real Time PCR and immunoblotting, *Perlecan *expression was found in all three cell lines with the highest levels present in the androgen sensitive LNCaP cell line (Figure 2A). We extended these findings by examining *Perlecan *expression with respect to tumor cell invasion and metastasis in an LNCaP tumor progression model. The LNCaP-derived cell line series (LNCaP, C4, C4-2, C4-2B) were derived from serial passage through nude mice [15,16]. The androgen sensitive parental LNCaP line is incapable of forming tumors in nude mice without stromal cell support. The C4 subline will form tumors when injected into castrated males, indicating that it is androgen insensitive, but will not metastasize. C4-2 is an androgen insensitive line that will metastasize, and the C4-2B subline is an androgen insensitive line that rapidly forms bone metastases. When *Perlecan *expression was assayed in the LNCaP series (Figure 2A) *Perlecan *RNA and protein was present in all the prostate cancer cell lines at levels lower than the androgen sensitive LNCaP cells. Thus all the androgen insensitive prostate cancer cell lines expressed lower levels of Perlecan RNA than he androgen sensitive cell line.

### Inhibition of Perlecan decreases prostate cancer cell proliferation in androgen sensitive and androgen insensitive tumor cells

To examine the direct effect of Perlecan on cancer cell growth we examined the ability of small interference RNA (siRNA) directed at *Perlecan *message to inhibit cell growth in the increasingly metastatic LNCaP cell line series LNCaP, C4, C4-2 and C4-2B. Proliferation assays demonstrated approximately equal decreases in BrdU incorporation for each cell line (Figure 2B). To evaluate the relationship between Perlecan and androgens on cancer cell growth we performed BrdU incorporation studies on the androgen sensitive LNCaP cells utilizing androgen blockade with bicalutimide (Casodex) with *Perlecan *siRNA or a scrambled siRNA control (Figure 2C). Independent application of *Perlecan *siRNA or androgen blockade resulted in 28% and 45% decreases in BrdU incorporation respectively. When combined, Perlecan siRNA and androgen blockade resulted in an additive effect with a 62% reduction.

### Perlecan correlates with Sonic Hedgehog expression

**Figure 3:**
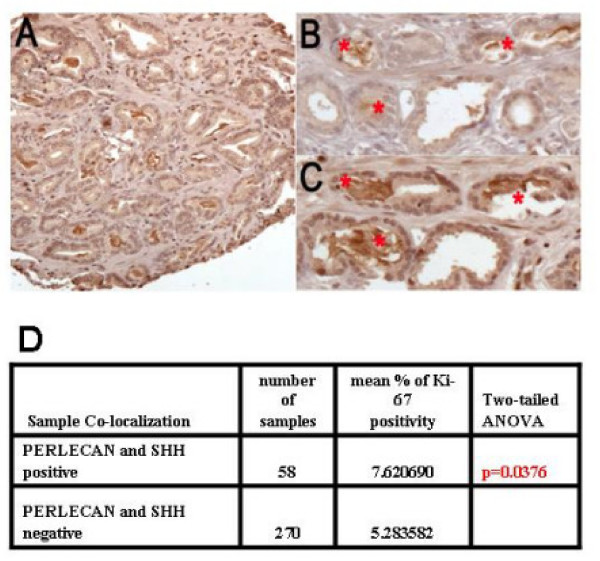
**Co-localization of Shh and Perlecan, and correlation with Ki-67 staining**. Immunohistochemistry for Sonic Hedgehog (A), demonstrating both weak cytoplasmic staining in prostate cancer epithelial cells and stronger intraluminal staining of secreted SHH. Co-localization of Perlecan (B) and Sonic Hedgehog (C) in consecutive sections of prostate carcinoma. Examples of co-localization of the secreted proteins in gland lumens are highlighted (red asterisks). All histologic images originally photographed at 400 × magnification. Significant co-localization of Perlecan and SHH staining was associated with higher cellular proliferation rates as indicated by Ki-67 nuclear staining by immunohistochemistry (D).

Since androgen signaling and Perlecan effects on tumor cell proliferation are independent, we asked what other signaling pathway Perlecan might be modulating to support prostate cancer cell growth. We and others have recently shown that Sonic Hedgehog regulates prostate cancer cell growth [6-9,17]. Since Perlecan has been implicated in Hedgehog signaling in *Drosophila *[5], we examined the correlation and interaction of Perlecan with Sonic Hedgehog in prostate cancer samples. Using sequential slides from tissue microarrays we compared the staining patterns for Perlecan and Sonic Hedgehog (Figures 3A–3C). Co-localization of Perlecan and Sonic Hedgehog staining was noted in a significant number of tumors, while luminal Sonic Hedgehog was not observed in normal prostate controls. In addition, co-localization of both Perlecan and Sonic Hedgehog correlated with increased tumor cell proliferation as shown by Ki-67 (PCNA) staining (Figure 3D). Our previous studies [7] had examined expression of SHH pathway genes in six matched benign and tumor patient samples where we have also examined Perlecan mRNA or protein expression (Figure 1G, 1H). In four common samples where we observe up-regulation of Perlecan in tumor tissue, we previously detected up-regulation of *SHH*, *PTCH1 *and *GLI1 *(patients 945, 1854, 921 and 1866) suggesting a complete functional pathway in these tumors. In two common samples where we observe decreased *Perlecan *mRNA levels, we previously saw decreased *SHH *expression (patients 829 and 887). Thus in individual patients, tumor expression of *Perlecan *and *SHH *are correlated, in agreement with the co-localization of Perlecan and SHH in tissue microarrays.

### Inhibition of Perlecan blocks Sonic Hedgehog signaling in cancer cells

**Figure 4:**
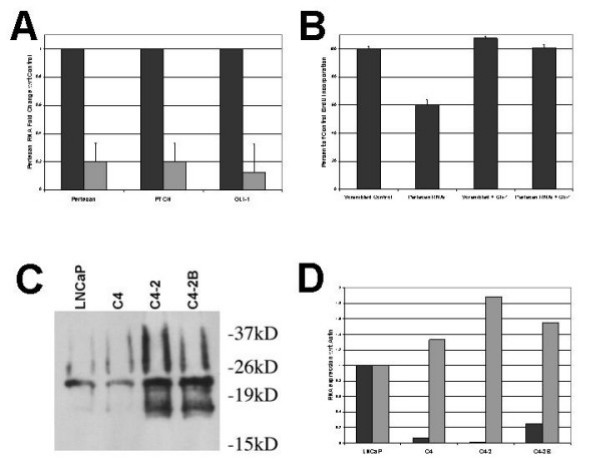
**Perlecan and the SHH-GLI1 pathway**. A. Decreased Perlecan and SHH signaling in *Perlecan *RNAi treated LNCaP cells. Expression of *Perlecan*, and the SHH signaling molecules *PTCH1 *and *GLI1 *as determined by Real Time PCR. Black columns represent control samples, Grey columns represent Perlecan RNAi treated cells. All expression normalized to β-*actin *levels. Real Time PCR studies were run with an n = 9. Error bars indicate standard deviation. B. Gli-1 transfection restores BrdU Proliferation in Perlecan RNAi treated cells. Percent BrdU incorporation normalized to levels of BrdU incorporation in control (scrambled RNAi treated) cells. BrdU analysis was done with n = 6. Error bars indicate standard deviation. C. Immunoprecipitation with anti-Perlecan antibody pulls down SHH. Co-immunoprecipitation of SHH and Perlecan from equal amounts of medium conditioned by 80% confluent cells. Size marker is indicated. Due to modifications, mature SHH runs as an approximately 22 kD band. Note the increased amount of bound SHH in the C4-2 and C4-2B cell lines. D. Relative expression of the SHH pathway components in LNCaP series cells. Black columns represent SHH mRNA, grey columns represent PTCH mRNA, with expression presented as ratios with respect to expression in LNCaP cells. While *SHH *is lower, *PTCH *is higher in the androgen insensitive metastatic cell lines C4-2 and C4-2B compared to LNCaP. All mRNAs by QRT-PCR were normalized to Beta-actin.

To investigate whether Perlecan is directly involved in modulating SHH signaling we examined the effect of *Perlecan *siRNA on expression of *PTCH1 *and *GLI1, *transcriptional targets of the SHH-GLI pathway [18] in LNCaP cells. Real-Time PCR analysis of *Perlecan *siRNA treated cells revealed the expected 80% decrease in Perlecan RNA, along with an 80% decrease in the level of *PTCH1 *expression and a 90% decrease in *GLI1*expression compared to controls (Figure 4A). A similar decrease in Perlecan protein levels in Perlecan siRNA treated LNCaP cells compared to control siRNA was noted (data not shown). These results demonstrate that Perlecan is required in androgen sensitive prostate cancer cells to achieve maximal SHH signaling activity.

Given that Perlecan has been shown to modulate the signaling of multiple growth factors including FGF2, FGF10 and VEGF, we asked if the reduction of prostate cancer cell growth in Perlecan siRNA treated cells was a result of decreased SHH signaling. If the decreased BrdU incorporation was due to inhibition of SHH signaling, then expression of the SHH downstream effector *GLI1 *should rescue the effects of Perlecan siRNA treatment. LNCaP cells were simultaneously transfected with Perlecan siRNA and an expression vector for *GLI1 *and their proliferation compared to that of controls transfected only with Perlecan siRNA (Figure 4B). As we observed earlier, transfection of Perlecan siRNA alone resulted in a drop in BrdU incorporation compared to controls. When Perlecan RNAi and the GLI1 expression vector were co-transfected, the percentage of BrdU labeling returned to control levels. Transfection of the *GLI1 *expression vector alone did not appreciably change LNCaP cell proliferation. This demonstrates that the major role of Perlecan in LNCaP cells is to maintain levels of SHH signaling.

### Perlecan forms a complex with Sonic Hedgehog

**Figure 5:**
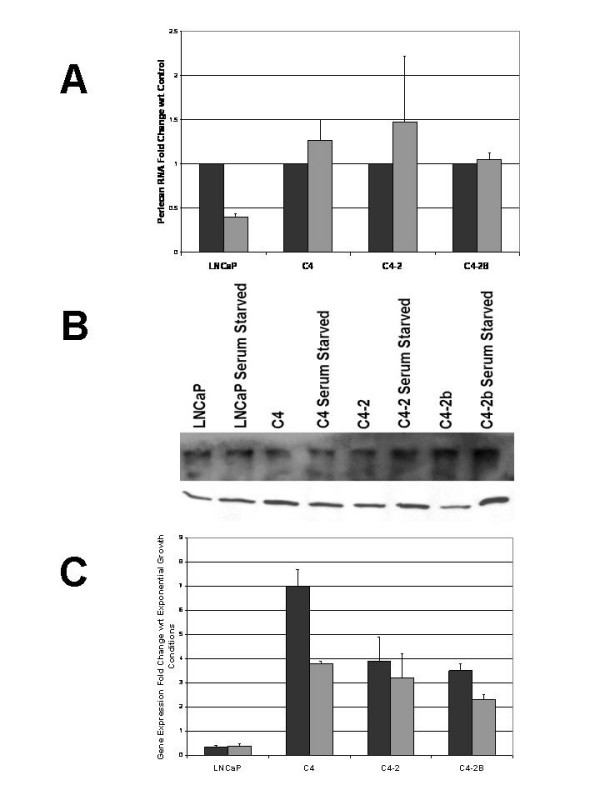
**Perlecan function under androgen and growth factor limitation**. A. Minimal changes in Perlecan mRNA levels in LNCaP-derived cell lines upon serum starvation. RealTime PCR analysis of Perlecan mRNA levels presented as fold increase in Perlecan under normal (black bars) or starved (grey bars) growth conditions. While Perlecan mRNA is decreased in LNCaP, all other cell lines demonstrate no change in Perlecan mRNA levels. B. Top Panel: No change in Perlecan protein levels upon serum starvation. Agarose based western blots from protein extracts derived from exponentially growing or serum starved LNCaP, C4, C4-2, and C4-2B cells. No significant differences are noted in protein levels between the cell lines or under the differing conditions. Bottom Panel: Equivalent amounts of the same samples loaded on traditional SDS-PAGE and probed for GAPDH as a loading control. C. Increases in expression of *SHH *and Gli-1 mRNA upon serum starvation. RealTime PCR analysis of *SHH *(black bars) and *GLI1 *(grey bars) as increased fold change compared to normal growth conditions. Gene expression determined by All Real Time PCR with an n = 9 and normalized to Beta-actin. Error bars indicate standard deviation.

Finally, we asked how Perlecan might affect signaling by SHH. Previously, we had demonstrated that Perlecan from flies or mice forms a complex with Hedgehog [5]. To test for a tumor cell complex containing both Perlecan and SHH we performed co-immunoprecipitation studies from the LNCaP series (Figure 4C). Perlecan-SHH complexes were detected in the conditioned medium of all cell lines under normal growth conditions. The mature Sonic Hedgehog protein was identified by Western blotting in all protein extracts precipitated with anti-Perlecan antibodies but not from extracts precipitated with control antibodies. Increased amounts of SHH-Perlecan complexes were detected in C4-2 and C4-2B, the two metastatic cell lines. The level of Perlecan protein does not change appreciably in the LNCaP series (Figure 5B), while the levels of *SHH *mRNA decrease across the series with increasing metastatic potential (Figure 4D). The presence of higher levels of SHH bound to Perlecan in the C4-2 and C4-2B cells when the levels of Perlecan protein are similar across the cell lines suggests increased binding of SHH to the available Perlecan. The increased amount of bound SHH is apparently functional, as Real-Time PCR studies indicate a relative increase in *PTCH1 *expression with respect to *SHH *in C4-2 and C4-2B when compared to LNCaP (Figure 4D). Taken together, the results of our expression, inhibition, and biochemical studies link *Perlecan *expression and function to SHH-GLI pathway activity in advanced prostate cancer cells.

### Tumor cells maintain *Perlecan *under poor androgen/growth factor conditions

The LNCaP series showed a large decrease in BrdU incorporation in response to Perlecan siRNA, indicating Perlecan based growth dependence under normal conditions regardless of their tumorigenic or metastatic potential. Our tissue microarray studies showed a correlation between Perlecan/SHH co-localization and both higher Gleason grade and stronger Ki-67 staining, suggesting that more aggressive or metastatic cells are more likely to use Perlecan-mediated SHH signaling. Since rapidly growing tumors tend to create microenvironments depleted of growth factors we asked if growth factor/androgen depletion via serum starvation would trigger the upregulation of *Perlecan *in an effort to more effectively use limiting growth factors such as SHH. In the parental LNCaP cell line, Perlecan mRNA levels decreased upon serum starvation (Figure 5A). However, the androgen insensitive C4, C4-2 and C4-2B lines maintained or increased their levels *Perlecan *expression upon serum starvation. Immunoblotting for Perlecan protein confirms these results for the cell lines under normal and serum starvation conditions (Figure 5B). We then asked if the expression of *Perlecan *in more metastatic lines under poor growth conditions correlated with SHH signaling activity. Real-Time PCR analysis for mRNA expression of *SHH *and the SHH response gene *GLI1 *upon starvation (Figure 5C) demonstrated that while expression of both *SHH *and *GLI1 *dropped in the LNCaP cell line, expression of both genes increased in the more tumorigenic and metastatic cell lines. Thus the level of *GLI1 *expression correlates with changes in *Perlecan *expression upon serum starvation in the LNCaP series (Figure 5A). This suggests that tumor cells such as C4, C4-2 and C4-2B that are capable of forming tumors and/or metastasizing without stromal support maintain a high level of SHH signaling under adverse growth conditions by maintaining high levels of *Perlecan *and *SHH *expression.

## Discussion

### Perlecan, a candidate oncogene for the CAPB locus

Using a bioinformatics based approach we identified Perlecan as a candidate oncogene involved in both prostate cancer and glioblastoma multiforme based on its genetic association with the CAPB locus at 1p36. Here we demonstrate Perlecan's expression and functional role in prostate cancer, and link it to the Sonic Hedgehog pathway known to be involved in glial tumorigenesis [19]. Thus from genetic mapping, physiological, and expression data there is evidence to suggest that Perlecan is a strong candidate for the CAPB oncogene. The results of interference with Perlecan function demonstrate that this proteoglycan is required for the growth of prostate cancer cells, extending its previously described roles in melanoma, colon, and lung cancer [20-22] and emphasizing Perlecan's role in multiple tumor types. Of note, genetic mapping studies have also identified a link between familial melanoma and 1p36, providing another link between Perlecan and tumorigenesis [23].

### Perlecan's regulation of growth factors and the link to Sonic Hedgehog

As Perlecan has been shown to bind a variety of growth factors in different tumors, the question as to which growth factor is being modulated in prostate cancer arose. Sonic Hedgehog has been associated with brain tumors and melanomas, two tumors with known genetic links to 1p36, where *Perlecan *is located [3,23]. Sonic Hedgehog has recently been linked to prostate cancer through a variety of studies [6]. We have demonstrated an increased frequency of Sonic Hedgehog positivity in prostate cancer tissue microarrays, and that Sonic Hedgehog signaling regulates tumor cell growth in both primary prostate tumor samples and prostate cancer cell lines [7]. High levels of Sonic Hedgehog activity, as monitored by *PTCH1*, *GLI1 *or *HIP *expression, are present in all metastatic prostate cancer samples that have been tested [8,9]. In fact, high levels of *PTCH1 *and *HIP *expression correlate with high (8–10) Gleason scores [8] where we have observed *Perlecan *expression. Furthermore, activation of the Sonic Hedgehog pathway by expression of *Gli *in the low metastatic potential rat AT2.1 cell line produced highly metastatic behavior, suggesting that high level activation of the Sonic Hedgehog pathway determines metastatic behavior [9]. Finally, Sonic Hedgehog promotes the growth of LNCaP derived xenograft tumors in mice [17]. We examined the potential of Perlecan to regulate Sonic Hedgehog signaling in tumors. The importance of heparan sulfate proteoglycans for Sonic Hedgehog signaling has been demonstrated in neural development, as mutations in the heparan sulfate binding site on Sonic Hedgehog causes decreased Sonic Hedgehog-driven proliferation [24]. In *Drosophila*, mutations in either Perlecan, or heparan sulfate synthesis or modification genes, greatly perturb Hedgehog signaling efficiency by affecting Hedgehog transport and binding [5,25-27]. Here we extend these findings in development to neoplasia by demonstrating that Sonic Hedgehog both co-localizes and directly binds to Perlecan in tumors, and that Sonic Hedgehog signaling occurs through Perlecan. This links Perlecan to the Sonic Hedgehog-Patched-Gli signaling pathway involved in prostate cancer [6], where Perlecan acts to modulate the effects of Sonic Hedgehog. As the Sonic Hedgehog signaling pathway has been linked to multiple tumor types including prostate, stomach, brain, and skin tumors [6] this evidence suggests a more general role for Perlecan in tumor regulation and tumorigenesis. We have surveyed a variety of tumor types and found SHH and Perlecan co-localization in a number of these, such as squamous cell carcinomas and adenocarcinomas of various origins along with tumors deriving from areas of normal *Perlecan *expression such as chondrosarcomas and osteosarcomas (data not shown).

### Perlecan in familial versus sporadic prostate cancers

We have demonstrated a positive correlation between Perlecan immunostaining and prostate tumors, in particular for high Gleason score tumors (Table 1). While genetic mapping studies make Perlecan an excellent candidate for the CAPB oncogene, our clinical validation has been performed on prostate samples without information regarding their familial prostate cancer history. Due to the rarity of families with familial brain and prostate tumors, it is most likely that the tumors studied do not represent CAPB kindreds. The suggested role of Perlecan in up-regulating Sonic Hedgehog signaling in sporadic prostate tumors, combined with its association with a prostate cancer genetic susceptibility locus, places Perlecan among a small group of genes with links to both familial and sporadic prostate cancers. This dual placement implies that Perlecan is part of a common oncogenesis pathway that both familial and sporadic tumors may traverse during oncogenesis. Of note, other members of the Sonic Hedgehog pathway, namely *SU(FU)*, *GLI1 *and *SMOH *also map to areas implicated in familial genetic studies (reviewed in [6]) and are up-regulated in studies of sporadic prostate cancer tumors [7-9]. Thus combining genetic analyses with evaluation of spontaneous tumors may allow us to identify the common pathways for carcinogenesis.

### Perlecan's role in prostate tumor growth: selective growth advantage for aggressive tumor cells under low androgen and/or growth factor conditions

High levels of Perlecan protein correlate significantly with aggressive, highly proliferating prostate tumors in our tissue microarrays and are also up-regulated in aggressive tumors from individual patients. Yet Perlecan is not present or overexpressed in every tumor or even in every metastatic site of tumor spread. While this result is not surprising considering the heterogeneity of neoplasia, it does suggest that subsets of tumors may utilize Perlecan signaling in specific situations. This correlation is demonstrated in the varied responses of the LNCaP-derived prostate cancer cell lines under poor growth conditions. In these situations *Perlecan *expression is maintained in the C4, C4-2, and C4-2B cell lines capable of forming stroma-independent tumors while the LNCaP parental line requires stromal support to form tumors and cannot maintain the Perlecan specific growth advantage [16]. This trait suggests a survival benefit to the more tumorigenic and metastatic tumor cells. Under poor growth conditions where low androgen and growth factor concentrations are present, the increased presence of Perlecan and its ability to concentrate growth factors would provide a survival advantage for tumor cells until a more suitable microenvironment can be found. In fact, our studies show that relative up-regulation of Perlecan expression by the more metastatic lines during serum starvation allowed them to maintain their levels of SHH stimulation, while the relative down-regulation of Perlecan expression in LNCaP resulted in decreased SHH signaling activity. Even under normal growth conditions, the more metastatic cell lines were able to form more Perlecan-SHH complexes and obtain greater SHH stimulation. Thus in the changing tumor microenvironment the more metastatic tumor cells have a choice of pathways (androgen, Perlecan-SHH) that can be modified or modulated to maintain tumor growth.

**Figure 6:**
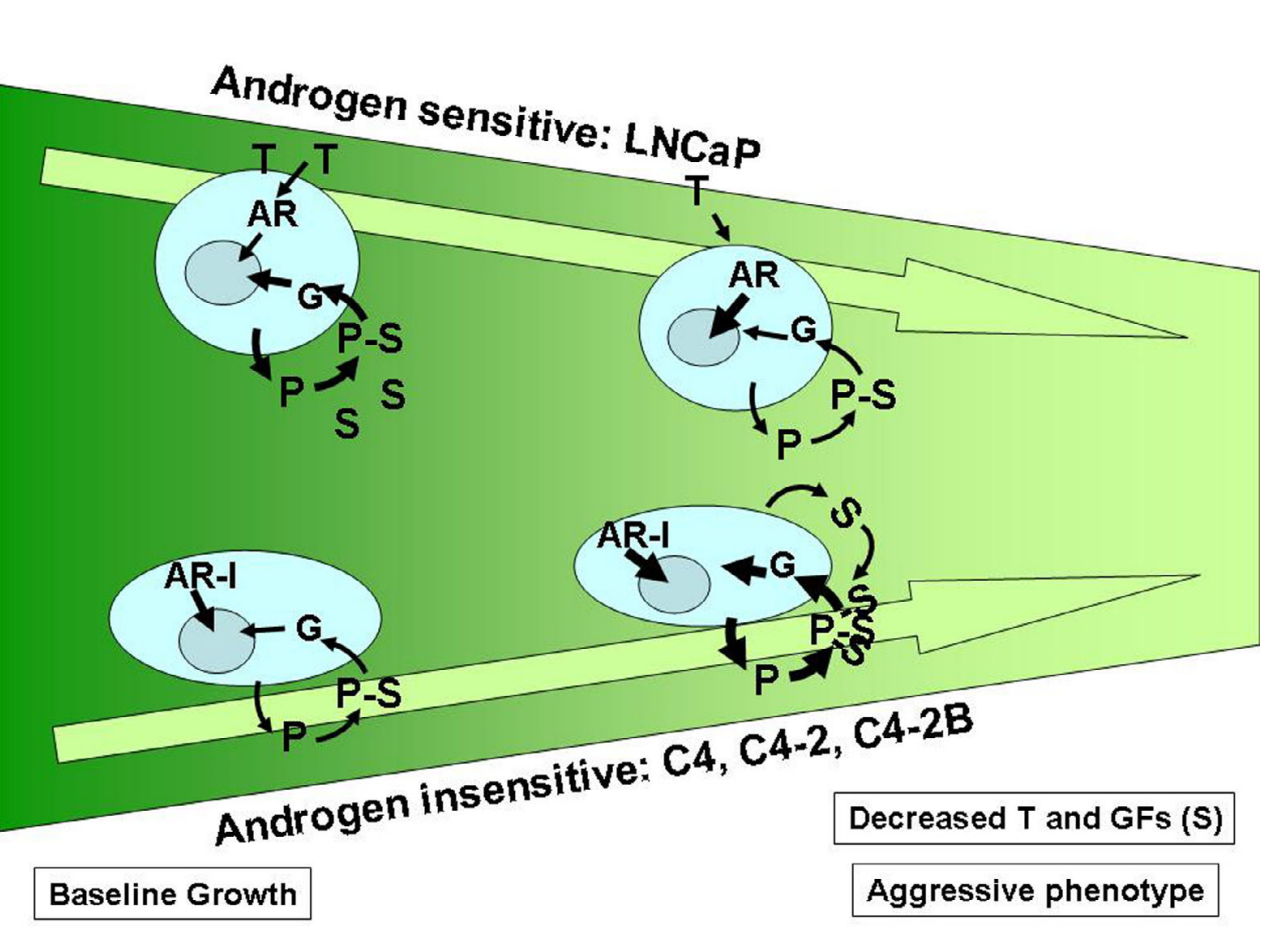
**Modulation of androgen and Perlecan regulated Sonic Hedgehog signaling**. As changes occur to the tumor microenvironment, prostate cancer cells modulate their use of both androgen and Perlecan mediated Sonic Hedgehog signaling. The use of androgen (T) occurs via the androgen receptor (AR). Perlecan (P) is produced, binds Sonic Hedgehog (S) and signals through the Gli (G) proteins. The heaviness of each arrow indicates relative signaling strength (gene expression, complex formation). Androgen sensitive cells (LNCaP) utilize both androgen and Perlecan-SHH signaling under normal conditions, but decrease Perlecan-SHH signaling under poor growth conditions. In contrast aggressive androgen insensitive cells (C4, C4-2, C4-2B) utilize both pathways, and upregulate the Perlecan-SHH signaling under poor growth conditions. This may occur through increased SHH binding affinity to Perlecan.

Heparan sulfate proteoglycans such as Perlecan have been shown to bind growth factors and may act as reservoirs or co-receptors for many growth factors [4]. Thus increasing Perlecan levels under growth factor limiting conditions such as within an inadequately vascularized tumor would be beneficial to a tumor cell. We propose that Perlecan may sustain the growth of nutrient starved prostate cancer cells in rapidly spreading tumors by amplifying their sensitivity and response to SHH signaling. These findings are summarized in a model of Perlecan action (Figure 6); in microenvironments with decreased growth factors and androgen, such as those encountered by rapidly growing tumors, Perlecan provides a secondary pathway for growth through SHH. This is used in both the androgen responsive and androgen insensitive aggressive tumor cells. Based on this model, one would hypothesize that chemotherapeutic treatments that simultaneously target both the androgen and the Perlecan-mediated Sonic Hedgehog pathways would provide the best control of androgen sensitive aggressive prostate cancer.

### Perlecan as a global regulator of growth factor action

While we have demonstrated that Sonic Hedgehog is critical to Perlecan-dependent cancer cell growth, other growth factors may also be regulated through Perlecan at different times or in different clinical stages. Recent results [13] suggest that Perlecan may regulate the activity of different growth factors during metastasis to bone. Thus the true role of Perlecan may not be regulating a single growth factor, but its ability to allow the tumor cell to adapt to differing tumor microenvironments by facilitating the signaling of different growth factors. If this is shown to be true, Perlecan may be an excellent target for drug targeting, with tumor specific targeting achieved through the selective blocking of specific growth factor binding sites on Perlecan.

### Perlecan function in metastasis, a role in the bony matrix

Perlecan is secreted by tumor cells, but is also present in specific stromal microenvironments in the body. This may affect a tumor's propensity to spread to specific sites. We have shown here that prostate cancer maintains Perlecan expression when it spreads to the lung or liver, but is less likely to do this in the soft tissue or lymph nodes. Maintaining or finding "Perlecan rich" sites may explain the propensity of tumors to home to specific sites during metastatic spread. A specific example of a Perlecan rich site would be the bone extracellular matrix, a major site for prostate cancer metastasis. In these sites Perlecan plays a role in normal bone formation and regulation through the modulation of growth factors utilized by osteoblasts [28-30]. Recent studies using the bone-targeted prostate cancer line C4-2B show that Perlecan is required for development of metastases through the modulation of growth factors, and leads to efficient tumor growth and vascularization [13]. Thus it appears that the presence of Perlecan in the bony matrix may help explain the tropism of prostate cancer to the bony matrix. Use of Perlecan as a drug target may prove advantageous by blocking bone metastasis and its associated morbidity. Lastly, Perlecan, as a secreted protein, may prove to be a useful biomarker for metastatic prostate cancer as well as a marker of either the risk or detection of tumor metastasis to bone since it can be easily detected in urine or serum samples, respectively.

## Methods

### Bioinformatics based analysis for candidate genes in the CAPB region

The 1p36 region, as defined by the chromosomal basepair data present in the human genome build 16 from the UCSC Genome Browser datasets, was searched for defined genes as identified in the NCBI LocusLink database. This search identified 5,108 expressed exons comprising 659 identified transcripts and 619 defined genes. Using text mining we searched a dataset of 3,737 prostate cancer genes as defined by co-localization of the gene name based on a hand annotated list from LocusLink and the words "prostate cancer" in MEDLINE. From this dataset 14 genes in the 1p36 region had been described in prostate cancer studies. A second text mining search we identified 15 genes in the CAPB region that also had been described in studies of the brain. None of the genes in the brain or prostate cancer text mining datasets were common. We then focused our examination on CAPB region genes with associated data in brain studies, and prostate and prostate cancer expression data from the Cancer Genome Anatomy Project (CGAP) along with cDNA microarray expression data generated in our laboratory for the prostate cancer cell lines LNCaP, DU-145, and PC3. A comparison of these datasets revealed three genes, EPHA2, HSGP2, and CAP2B, with data in both brain research studies and expression in the prostate cancer or the precancerous change high grade prostatic intraepithelial neoplasia. Of these three genes, HSPG2 also was contained within our prostate cancer cell line cDNA expression datasets, with increased levels of expression in the derived invasive sublines of PC3 when compared to a derived non-invasive subline.

### Prostate samples and tissue culture

LNCaP, PC3 and DU-145 cell lines were obtained from ATCC and grown under standard conditions. The LNCaP series LNCaP, C4, C4-2 and C4-2B were obtained from Dr. L. Chung. All primary prostate tumors were obtained by MWD using approved protocols with informed consent on the part of the subjects.

### Real Time PCR on cell line RNA samples

Total RNA isolated from cell lines using Trizol and then further purified using the RiboPure kit (Ambion). Purified RNA was digested with DNAse (Invitrogen), and analyzed using the SYBER Green system according to manufacturers protocols (Applied Biosystems) on an ABI Prism 7700 machine. Each sample was run in triplicate at three different concentrations. Primers were designed using Primer Express software and are available upon request. Fold increase/decrease comparisons were calculated using the delta-delta CT method.

### Tissue microarray and immunohistochemistry

Upon institutional review board approval, a tissue microarray was prepared from 288 radical prostatectomy cases present at the Medical College of Wisconsin. A second tissue microarray was prepared from samples collected under approved protocols at the University of Pittsburgh Medical Center. 0.6 mm cores were arrayed and 5 um sections processed. Benign tissue, high-grade prostatic intraepithelial neoplasia, or invasive tumor tissue were identified by MWD or RD by high molecular weight cytokeratin staining (CK903 Ab, DAKO). A third tissue microarray was prepared from samples collected under approved protocols as part of the rapid autopsy program at the University of Michigan. For microarray samples, a common antigen retrieval procedure was carried out. Slides were processed for Perlecan or SHH and developed with HRP conjugated secondary antibodies and DAB substrate. For a portion of the tissue microarray anonymous de-identified pathologic and outcomes data were available. Individual cores were examined as duplicates and staining correlated using Chi-squared, Fisher's Exact or two-tailed ANOVA analyses.

### Transfection and proliferation assays

Purified and desalted siRNAs were purchased from Ambion as a proprietary non-validated *Perlecan *siRNA and a scrambled siRNA control. SiRNA and *GLI1 *expression vector transfections were carried out with Lipofectamine 2000 (Invitrogen) as described by the manufacturer and effects measured after 72 hours. Casodex was used in cell cultures as described previously. Immunocytochemistry on cell lines was carried out using with anti-BrdU (Research Diagnostics or Becton-Dickinson) and HRP-conjugated secondary antibodies (Boehringer Mannheim) using standard techniques.

### Protein extracts, Western blotting and immunoprecipitations

Normal and tumor tissue from the same patients were obtained as described below following approved protocols. Sections were assessed pathologically by a urologic pathologist (MWD) to determine areas of normal and tumor tissue. Samples were microdissected and total protein isolated. Proteins were also isolated from cultured medium from cell lines grown under normal or serum starved conditions. Proteins were run on a 1.6% agarose gel, blotted and probed for Perlecan (Chemicon). Equal samples were loaded onto a standard SDS-PAGE gel, blotted and probed for GAPDH (Santa Cruz) as a loading control. Equal amounts of conditioned medium from equivalently confluent cell lines were immunoprecipitated with an anti- Perlecan or unrelated control antibody, the resulting complex run on denaturing SDS-PAGE, and the presence of SHH verified by immunoblotting (Santa Cruz).

## Competing interests

Drs. M. W. Datta and S. Datta declare competing interests due to a patent application.

## Authors' contributions

M.W.D. provided human samples and was responsible for pathological evaluation of tissue, interpretation of tissue microarrays, experimental design for both cell culture and tissue studies, writing of manuscript and providing funding.

A.M.H. was responsible for Real-Time PCR and Western analyses.

M.J.S. carried out Perlecan siRNA studies and Perlecan-SHH Co-IP.

A.J.K. carried out IHC of the tissue microarrays for Perlecan.

A.M.D assisted the IHC of the tissue microarrays for Perlecan.

R.D. provided prostate tumor microarrays and associated clinical data.

R.S. provided metastatic tumor microarrays and associated clinical data.

C.F.C. provided assistance and advised in the staining interpretation and use of Perlecan antibodies.

A.B. provided RealTime PCR data.

S. D. was responsible for Perlecan-SHH Co-IP, IHCs for Perlecan and SHH, developing Western technique for Perlecan, experimental design for cell culture studies and overall experimental design, writing of manuscript and providing funding.
